# Identification of Novel Adenylyl Cyclase 5 (AC5) Signaling Networks in D_1_ and D_2_ Medium Spiny Neurons using Bimolecular Fluorescence Complementation Screening

**DOI:** 10.3390/cells8111468

**Published:** 2019-11-19

**Authors:** Trevor B. Doyle, Brian S. Muntean, Karin F. Ejendal, Michael P. Hayes, Monica Soto-Velasquez, Kirill A. Martemyanov, Carmen W. Dessauer, Chang-Deng Hu, Val J. Watts

**Affiliations:** 1 Medicinal Chemistry & Molecular Pharmacology, Purdue University, West Lafayette, IN 47906, USA; doyle34@purdue.edu (T.B.D.); kejendal@purdue.edu (K.F.E.); hayes158@purdue.edu (M.P.H.); msotovel@purdue.edu (M.S.-V.); hu1@purdue.edu (C.-D.H.); 2 Department of Neuroscience, The Scripps Research Institute, Jupiter, FL 33458, USA; BMuntean@scripps.edu (B.S.M.); Kirill@scripps.edu (K.A.M.); 3 Department of Integrative Biology & Pharmacology, McGovern Medical School, University of Texas Health Science Center, Houston, TX 77030, USA; Carmen.W.Dessauer@uth.tmc.edu; 4 Purdue Institute of Drug Discovery, Purdue University, West Lafayette, IN 47906, USA; 5 Purdue Institute for Integrative Neuroscience, Purdue University, West Lafayette, IN 47906, USA

**Keywords:** adenylyl cyclase, AC5, BiFC, cAMP, dopamine, PP2A, striatum, NAPA

## Abstract

Adenylyl cyclase type 5 (AC5), as the principal isoform expressed in striatal medium spiny neurons (MSNs), is essential for the integration of both stimulatory and inhibitory midbrain signals that initiate from dopaminergic G protein-coupled receptor (GPCR) activation. The spatial and temporal control of cAMP signaling is dependent upon the composition of local regulatory protein networks. However, there is little understanding of how adenylyl cyclase protein interaction networks adapt to the multifarious pressures of integrating acute versus chronic and inhibitory vs. stimulatory receptor signaling in striatal MSNs. Here, we presented the development of a novel bimolecular fluorescence complementation (BiFC)-based protein-protein interaction screening methodology to further identify and characterize elements important for homeostatic control of dopamine-modulated AC5 signaling in a neuronal model cell line and striatal MSNs. We identified two novel AC5 modulators: the protein phosphatase 2A (PP2A) catalytic subunit (PPP2CB) and the intracellular trafficking associated protein—NSF (N-ethylmaleimide-sensitive factor) attachment protein alpha (NAPA). The effects of genetic knockdown (KD) of each gene were evaluated in several cellular models, including D_1_- and D_2_-dopamine receptor-expressing MSNs from CAMPER mice. The knockdown of PPP2CB was associated with a reduction in acute and sensitized adenylyl cyclase activity, implicating PP2A is an important and persistent regulator of adenylyl cyclase activity. In contrast, the effects of NAPA knockdown were more nuanced and appeared to involve an activity-dependent protein interaction network. Taken together, these data represent a novel screening method and workflow for the identification and validation of adenylyl cyclase protein-protein interaction networks under diverse cAMP signaling paradigms.

## 1. Introduction

The striatum is the principle input nucleus of the basal ganglia and relies on the integration of diverse cortical and thalamic inputs to coordinate and control movement [1]. The canonical model of basal ganglia function describes two dopaminergic neural circuits, the direct and indirect pathways, which originate from distinct populations of striatal medium spiny neurons (MSNs) and act in concert to process signals involved in movement [2]. The regulation of these two opposing circuits occurs through differential expression of opposing dopamine receptor subtypes, stimulatory (D_1_) and inhibitory (D_2_), in these neuronal populations [2]. The D_1_ dopamine receptor (D_1_R) expressing MSNs of the direct pathway project to and inhibit the substantia nigra reticulata (SNr) and globus pallidus to promote movement [3]. Conversely, the activation of D_2_ dopamine receptor (D_2_R) expressing MSNs of the indirect pathway project to the external segment of the globus pallidus to suppress movement [3]. The balanced signaling of these dopaminergic circuits relies on the precise cellular integration of receptor-mediated signaling through the enzyme adenylyl cyclase (AC).

Adenylyl cyclase type 5 (AC5) is the principal isoform expressed in striatal MSNs and is known to be a critical regulator of the integration and control of midbrain dopaminergic signaling through the production of the second messenger cAMP [4,5]. Adenylyl cyclases exhibit three general modes of regulation; the acute activation of Gα_s_/_olf_ coupled receptors, such as the D_1_R, stimulates AC activity, increasing the production of cAMP [6,7]. In contrast, the acute activation of Gα_i/o_-coupled receptors, such as the D_2_R, results in the inhibition of AC, decreasing cAMP [8,9,10]. However, chronic activation of Gα_i/o_-coupled receptors results in a paradoxical enhancement of cAMP production. This compensatory mechanism of enhanced AC activity following prolonged inhibition is known as heterologous sensitization or supersensitization [7,11].

The precise spatial and temporal control of cAMP signaling within a cellular compartment is governed by the composition of local regulatory protein networks [12,13,14]. The composition and localization of these dynamic regulatory complexes are dependent upon a variety of interdependent cellular signals, including G protein-coupled receptor (GPCR) activity [15,16,17]. Time-resolved analysis of GPCR protein networks has demonstrated that chronic receptor activation can result in the formation of protein networks that are unique from those that occur following acute receptor activation [18]. While there is increasing evidence that receptor protein interaction networks are dependent on the duration of activation, there is little understanding of how AC protein interaction networks adapt to the dual pressures of integrating acute versus chronic and inhibitory vs. stimulatory receptor signaling in striatal MSNs.

**Figure 1:**
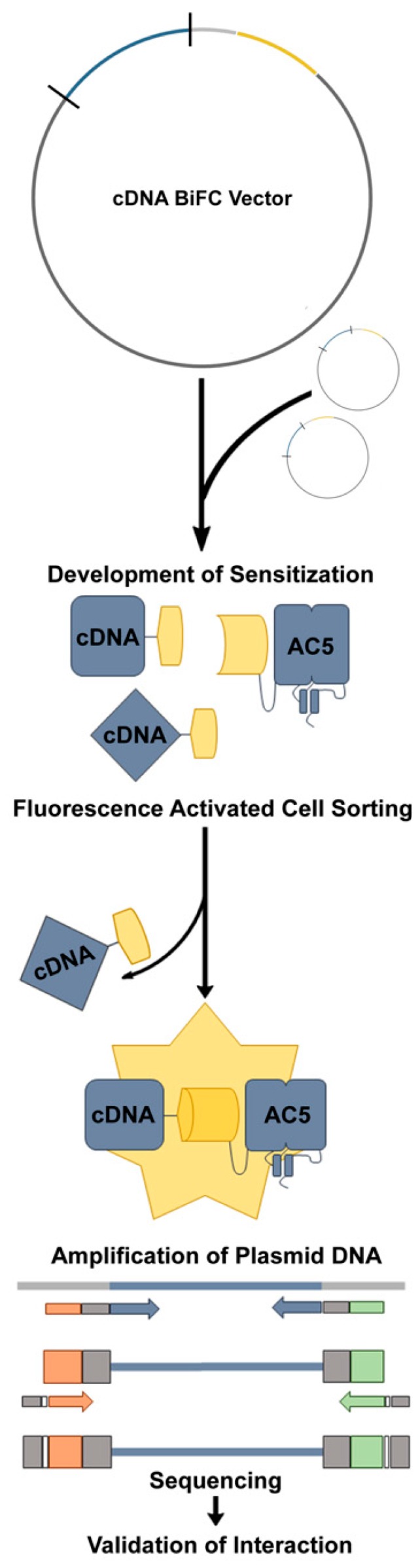
Screen workflow for the identification of adenylyl cyclase type 5 (AC5) interaction network partners using unique bimolecular fluorescence complementation (BiFC) tagged cDNA library. A unique human brain cDNA library was cloned into the BiFC plasmid, such that each cDNA would be expressed linked to the C-terminal fragment of Venus (i.e., cDNA-VC). The BiFC tagged cDNA library was transiently expressed into CAD VN-AC5/D_2L_, and the development of heterologous sensitization initiated. Fluorescence-activated cell sorting was used to identify and isolate cells that exhibited an AC5-protein network interaction, as determined by a BiFC fluorescent signal. Next-generation sequencing (NGS) was used to identify the potential interacting proteins from the complementing cDNAs, followed by the siRNA knockdown of identified genes to confirm their role in heterologous sensitization.

Here, we presented the development of novel bimolecular fluorescence complementation (BiFC)-based protein-protein interaction network screening methodology (Figure 1) to further identify and characterize elements important for homeostatic control of cAMP in striatal MSNs. To accomplish this, we developed a neuronal cellular model expressing an AC5 fusion construct containing a fragment of YFP and used a human cDNA library containing the complementary portion of YFP. We employed fluorescence-activated cell sorting (FACS) to detect and capture BiFC-positive cells under basal conditions, as well as following prolonged D_2_R activation, in a high-throughput manner. Next-generation sequencing (NGS) allowed for the retroactive identification of AC5 network interacting partners under the unique treatment conditions. Pathway analysis assisted in the identification of two novel AC5 modulators: the protein phosphatase 2A (PP2A) catalytic subunit (PPP2CB) and the intracellular trafficking associated protein—NSF (N-ethylmaleimide-sensitive factor) attachment protein alpha (NAPA). The effects of genetic knockdown of both genes were evaluated in several cellular models, including D1- and D2-dopamine receptors, expressing MSNs from CAMPER mice. The genetic knockdown of PPP2CB was associated with a reduction in acute and sensitized AC activity in each model tested, implicating PP2A is an important and persistent regulator of AC activity. In contrast, the effects of NAPA knockdown were more nuanced and appeared to involve in the activity-dependent protein interaction network. Together, these results demonstrated the utility of our newly developed BiFC-FACS-NGS screening paradigm in the identification of novel, physiologically relevant AC modulating proteins.

## 2. Materials and Methods

### 2.1. Cell Culture

Human embryonic kidney (HEK) 293 cells stably expressing the long isoform of the human D2 dopamine receptor (D2L) and human AC type 5 (HEK-AC5/D2L) were cultured in Dulbecco’s modified eagle medium (Life Technologies, Grand Island, NY, USA), supplemented with 5% bovine calf serum (Hyclone, Logan, UT, USA), 5% fetal clone I (Hyclone, Logan, UT, USA), 1% antibiotic-antimycotic 100x solution (Life Technologies, Grand Island, NY, USA). Stable cell clones were maintained in culture media containing 800 μg/mL G418 (InvivoGen, San Diego, CA, USA) and 2 μg/mL puromycin (Sigma Aldrich, St. Louis, MO, USA). A Cath a. differentiated (CAD) cell line was constructed to express the long isoform of the human D2 dopamine receptor (D2L) and human AC5 linked by the N-terminus to the N-terminal BiFC fragment of the Venus fluorescent protein (VN155-AC5) through a short alanine-rich, flexible linker CAD (VN-AC5/D2L cells). Stable cell clones were cultured in Dulbecco’s modified eagle medium (Life Technologies, Grand Island, NY, USA), supplemented with 5% bovine calf serum (Hyclone, Logan, UT, USA), 5% fetal bovine serum (Hyclone, Logan, UT, USA), 1% antibiotic-antimycotic 100x solution (Life Technologies, Grand Island, NY, USA). Stable cell clones were maintained in culture media containing 800 μg/mL G418 (InvivoGen, San Diego, CA, USA) and 2 μg/mL puromycin. All cell lines were maintained in a humidified incubator at 37 °C and 5% CO_2_.

### 2.2. BiFC Plasmid Construction and Validation

The BiFC plasmid vector used for cDNA library screening was synthesized by Genscript (Piscataway, NJ, USA). Briefly, a plasmid was constructed using the pcDNA3.1+ backbone. The complementary VC155 BiFC fragment was separated from an EcoRI restriction site by a 15 amino acid serine-glycine-glycine repeat flexible linker. To validate the function of the BiFC plasmids, AC5-interacting partners were cloned into the EcoRI restriction site, and the orientation was validated by sequencing. CAD VN-AC5/D_2L_ cells were plated in 6-well dish and cultured to 80–90% confluency, the culture media was replaced, and cells were transfected with BiFC plasmid controls using Lipofectamine 2000 according to the manufacturer’s recommendations. Images were collected 48 h after transfection using a Nikon A1 confocal microscope system and NIS-Elements 4.5 software (Nikon Instruments, Melville, NY, USA) and analyzed using ImageJ software (NIH, 1.47v, Bethesda, MD, USA). Line profile analysis was performed using NIS Elements 4.0 software (Nikon Instruments, Melville, NY, USA).

### 2.3. cDNA Library Transfection and Cell Treatment

Express Genomics cloned a unique human fetal brain cDNA library (Express genomics, Frederick, MD, USA), which contained a minimum of 1 × 10^6^ independent clones into the EcoRI restriction sites of the BiFC vector. The resulting BiFC-linked cDNA library was then validated by a cDNA insert sizing analysis. CAD VN-AC5/D_2L_ cells were seeded in four 100 mm culture dishes, then incubated at 37 °C and 5% CO_2_ in selection media until they reached approximately 80% confluency. Selection media was gently aspirated and replaced with CAD culture media lacking any selection agents. Fourteen micrograms of the cDNA library were transfected using Lipofectamine 2000 following the manufacturer’s recommended directions. Twenty-four hours following the cDNA library transfection, the media was gently aspirated, then replaced with culture media containing DMSO (vehicle) or 1 μM quinpirole treatment. All plates were returned to the incubator overnight. To remove cells, they were washed gently with warm PBS and dissociated using non-enzymatic cell dissociation buffer (Gibco, Grand Island, NY, USA). Cells were collected in a conical tube and pelleted by centrifugation at 800× *g* for 5 min. The supernatant liquid was aspirated, and the cell pellet was resuspended in a solution of warm Hank’s Balanced Salt Solution (HBSS) containing 10 mM HEPES and 2% BSA for FACS analysis.

### 2.4. Fluorescence-Activated Cell Sorting (FACS)

The fluorescence collection parameters for each FACS sort were first established using a sample of non-transfected cells to determine basal levels of fluorescence and scatter. For each treatment condition, cDNA library transfected cells exhibiting positive fluorescence above basal were collected. Flow cytometry and FACS analyses were performed using a BD FACS Aria II (BD Biosciences, San Jose, CA, USA). Excitation at 488 nm using an argon laser, 50LP filter with 525/50 emission was used for FACS sorts. Transfected cells were passed through a 40 μm cell strainer before FACS analyses. FACS analyses were run under 20 psi (sample/sheath) using a 100 μm nozzle. Sheath fluid was 1x PBS at pH 7, and the cells were sorted directly into a collecting tube containing HBSS with 0.01% HEPES and 1% BSA. The BiFC fluorescent-positive sorted cells were centrifuged at 800× *g* for 10 min, the supernatant was aspirated, and the remaining cell pellet was stored at −80 °C until use.

### 2.5. Plasmid Extraction and PCR

Cell pellets were removed from −80 °C and freeze fractured in liquid nitrogen to lyse cells. Plasmid DNA extraction and isolation was performed using a Spin Miniprep Kit (Qiagen, Germantown, MD, USA) according to the manufacturer’s protocol. The cDNA insert region of the extracted plasmid DNA was amplified and prepared for NGS using a two-step PCR approach. The 1st stage PCR primers contained a locus-specific sequence unique to the cDNA insert region of the BiFC plasmid, and an overhanging adapter sequence for binding the 2nd stage PCR primers. The 2nd stage PCR reaction added the adapters for Illumina sequencing, and a small sequence unique to each FACS sort and treatment to the amplified cDNA sequence. PCR products were cleaned between reactions using a DNA cleanup kit (Qiagen, Germantown, MD, USA) according to the manufacturer’s protocol. The PCR reactions were carried out using a T100 Thermal Cycler (BioRad, Hercules, CA, USA) in 50 μL volumes. The thermocycler program included a longer extension time to account for the unknown amplicon size and is outlined below. The final PCR products from each FACS sort were then pooled together for sequencing.

### 2.6. Library Preparation Illumina MiSeq Sequencing

Sequencing of the variable cDNA region amplicon library was done on the Illumina MiSeq platform at the Purdue University Genomics Core Facility, and 300 bp paired-end reads generated. After trimming the adaptor and primer sequences from the Illumina reads, the raw sequences of the cDNA library were compared against the reference human genome GRCh38. A list was compiled of the genes that appeared under each treatment condition, for all three independent FACS events. Genes that appeared in at least two of the three sorts were selected for further evaluation.

### 2.7. Cisbio HTRF cAMP Assay

cAMP levels were measured using the Cisbio HTRF cAMP dynamic-2 assay kit according to the manufacturer’s instructions. Cells growing in 100 mm plates had their culture media aspirated, were washed gently with phosphate-buffered saline, then dissociated with non-enzymatic cell dissociation buffer (Gibco, Grand Island, NY, USA). Cell suspensions were centrifuged at 500× *g* for 5 min, and the supernatant was aspirated. Cell pellets were resuspended in OptiMEM buffer, diluted as indicated, and 10 μL/well seeded into a white, flat bottom, tissue culture-treated 384-well plate (PerkinElmer, Shelton, CT, USA). Assay plates containing cells were briefly centrifuged at 100× *g* for 30 s and incubated in a humidified incubator at 37 °C and 5% CO_2_ for 1 h before receiving a specific treatment.

### 2.8. Reverse siRNA Transfection for qPCR and Western Blotting

Lyophilized siRNA was resuspended in 1x siRNA buffer (Dharmacon, Longmont, CO, USA) and diluted to 20 μM stocks, then further diluted to 0.5 μM in OptiMEM. Lipofectamine RNAiMAX was diluted in OptiMEM (9 μL/mL), and 320 μL of 0.5 μM siRNA was mixed with 1024 μL dilute RNAiMax, then added to a 6-well dish to incubate for 30 min at room temperature. Cells in culture had the media aspirated, were rinsed briefly with phosphate-buffered saline, then dissociated with non-enzymatic cell dissociation buffer. Cell suspensions were centrifuged at 500× *g* for 5 min, and cells were resuspended in OptiMEM containing 7.5% heat-inactivated fetal bovine serum (Hyclone, Logan, UT, USA). Cells were diluted, then 2.5 mL cell solution was added to the 6-well dish containing siRNA. Cells were incubated in a humidified incubator at 37 °C and 5% CO_2_ for 72 h before dissociation for qPCR or western blotting.

### 2.9. Quantitative PCR (qPCR)

Cells were washed in phosphate-buffered saline, then removed from plates using non-enzymatic cell dissociation buffer. RNA extraction was performed with the RNeasy mini kit (Qiagen, Germantown, MD, USA). Reverse transcription step was carried out using the iScript cDNA synthesis kit (BioRad, Hercules, CA, USA). RT-qPCR was completed using the SYBR Green Reagents kit according to manufacturer’s protocol (Thermo Scientific, Waltham, MA, USA) in 384 well qPCR plates (Dot Scientific, Burton, MI, USA) using 15 μL/well volumes. RT-qPCR was read using a ViiA 7 Real-Time PCR System (Thermo Scientific, Waltham, MA, USA). qPCR primers were purchased from Integrated DNA Technologies (Coralville, IA, USA).

### 2.10. Western Blotting

Unless otherwise specified, reagents were purchased from Sigma Aldrich (St. Louis, MO, USA). Anti Gα_s/olf_ antibody was purchased from Santa Cruz Biotechnology (Dallas, TX, USA). Anti-NAPA and anti-alpha tubulin antibodies were purchased from Novus biological (Littleton, CO, USA). Anti-PPP2CB antibodies were purchased from Abcam (Cambridge, MA, USA). Briefly, cells were washed with phosphate-buffered saline before being dissociated from the plate with non-enzymatic cell dissociation buffer and centrifuged at 800× *g* for 5 min. The cell pellet was resuspended in RIPA lysis buffer and incubated on ice for 30 min, then centrifuged to pellet the insoluble fraction. Protein samples were separated by SDS Page and transferred to the PVDF membrane. Membranes were blocked in 5% non-fat milk for 1 h at room temperature, the membrane was probed for the protein of interest with primary antibodies, diluted in phosphate-buffered saline (PBS) + 0.5% Tween20 (PBST) with 1% milk, by rocking overnight at 4 °C. The membrane was washed with PBST, then incubated with a secondary IRDye 680RD anti-mouse or IRDye 800CW anti-rabbit (LICOR Biotechnology, Lincoln, NE, USA) at 1:10,000 for 1 h at room temperature. To visualize the bands, the membrane was imaged on a LICOR Odyssey CLx (LICOR Biotechnology, Lincoln, NE, USA). Band intensities were quantified using ImageJ software (NIH, Bethesda, MD, USA).

### 2.11. Immunoprecipitation

Immunoprecipitation of AC activity was conducted, as previously described [17]. Briefly, non-transfected HEK AC5/D_2L_ cells were grown to 90% confluency in 10-cm dishes, and the cell lysis buffer (300 μL; 50 mM HEPES, pH 7.5, 1 mM EDTA, 1 mM MgCl_2_, 150 mM NaCl, 0.5% C_12_E_10_) was added to plates on ice. Cell lysates were collected, centrifuged at 13,000× *g*, and diluted before adding appropriate antibody and rotating overnight at 4 °C. Protein A agarose beads were added to each sample and rotated for 1 h at 4 °C before removing the supernatant and washing the beads. Samples for western blotting were resuspended in a 1x sample buffer and run as western blots, as described previously. Samples for AC assays were run, as described in supplemental methods.

### 2.12. Protein Expression and Purification

Gα_s_ was expressed as an N-terminally 6XHis tagged protein in BL21(DE3) *Escherichia coli*, essentially as described previously [19]. This protein has previously been shown to stimulate cAMP production in membrane fractions from cells overexpressing AC5 [20]. Colonies from a fresh transformation were selected and cultured at 37 °C and 250 rpm in Terrific Broth until OD600 of 0.5 was reached, at which time the cultures were induced with 30 μM IPTG and grown for 16 h at 18 °C. Bacterial cells were pelleted at 1000× *g* and resuspended in Buffer A (25 mM HEPES, 5 mM MgCl_2_, 150 mM NaCl, 5 mM β-mercaptoethanol, 20 μM GDP, pH 8) and supplemented with 1 mg/mL lysozyme and 0.01 mg/mL DNase I. Cell lysis was performed for 20 min on ice, and lysate was clarified by centrifugation at 30,000× *g*. The supernatant was then subjected to immobilized metal affinity chromatography using 5 mL His-Pur Ni-NTA resin (Thermo Scientific, Waltham, MA, USA) and eluted using Buffer A containing 200 mM imidazole and 1 mM NaCl. Gα_s_-containing fractions were identified via SDS-PAGE, pooled, and further purified by ion-exchange chromatography using a 1 mL HiTrap Q Sepharose column (GE Healthcare, Chicago, IL, USA) over a gradient of Buffer A containing 1 mM to 400 mM NaCl. Gα_s_-His containing fractions were snap-frozen in liquid nitrogen and stored at −80 °C until use. Nucleotide exchange was performed in 50 mM HEPES, 2 mM DTT, 250 mM GTPγS, and 1 mM MgCl_2_ for 20 min on ice, followed by 30 min at 30 °C, as described previously [20].

### 2.13. Lentivirus Production

Three sgRNA sequences targeting Napa (5′-CTTGAACATGTTCGCCGCTC-3′, 5′-AGATTGCTCATAGTGGGCGA-3′, 5′-TGCTGGGAACGCTTTCTGCC-3′) and Ppp2cb (5′-TCTCGCACAGCGTCCGCACT-3′, 5′-ATACGAACTACCTATTCATG-3′, 5′- CGCAATATTGTGATGCGCTC-3′) were designed using CHOPCHOP (version 3) [21]. Custom oligos (Integrated DNA Technologies, Coralville, IA, USA) were phosphorylated in vitro with T4 polynucleotide kinase (New England Biolabs, Ipswich, MA, USA), annealed by heating/cooling in a thermocycler, ligated into the BsmBI site of pSECC (Addgene # 60820) with T4 DNA Ligase (New England Biolabs, Ipswich, MA, USA), and plasmid was purified from Stbl3 *E. coli* (Thermofisher, Waltham, MA, USA). Lentiviral particles for each individual sgRNA construct and a scrambled control (5′-GCGAGGTATTCGGCTCCGCG-3′) [22] were generated by Lipofectamine Plus/LTX-mediated transfection of 293FT cells with pSECC, pCMV-VSV-G (Addgene #8454), pMDLg/pRRE (Addgene #12251), and pRSV-Rev (Addgene #12253). The supernatant containing viral particles was collected at 48 h post-transfection.

### 2.14. Primary Culture

As previously described [23], striata from pups at age P0 from homozygous CAMPER breeders (JAX 032205: C57BL/6-Gt(ROSA)26Sortm1(CAG-ECFP*/Rapgef3/Venus*)Kama/J) were dissected in ice-cold HBSS supplemented with 20% FBS, 4.2 mM NaHCO3, and 1 mM HEPES. Pooled striata were then washed in HBSS absent FBS and digested at 37 °C for 15 min in a pH 7.2 buffer containing (in mM): NaCl (137), KCl (5), Na2HPO4 (7), HEPES (25), and 0.3 mg/mL Papain (Worthington, Lakewood, NJ, USA). Striata were then washed three times with each of the following solutions: HBSS/FBS, HBSS, growth media (Neurobasal-A supplemented with 2 mM GlutaMAX, 2% B27 Supplement serum-free, and 1% PenStrep). Tissue dissociation was performed with a standard P1000 pipette in the presence of DNAse I (0.05 U/μL), cells were passed through a 40 μm cell strainer and plated on poly-D-lysine coated glass coverslips. Neuron cultures were maintained at 37 °C/5% CO_2_ in a humidified incubator, and half of the growth media was replaced every three days. Lentiviral transductions were performed by directly adding supernatant containing lentiviral particles. The animal studies were carried out in accordance with the National Institutes of Health guidelines and were granted formal approval by the Institutional Animal Care and Use Committee of the Scripps Research Institute (approved protocol #16-032).

### 2.15. Confocal Microscopy

As previously described [23], coverslips containing neuronal cultures were imaged under a Leica TCS SP8 MP confocal microscope by excitation of mTurquoise FRET donor with a 442 nm diode laser paired with simultaneous collection of 465–505 nm (mTurquoise FRET donor) and 525–600 nm (Venus FRET acceptor) bandpass emission filtration at 3.5 Hz. XYZ image stacks were acquired at 10 s intervals through a 25x objective lens. Quantification of fluorescence intensity was performed on neuronal cell bodies using ImageJ to calculate FRET from the inverse ratio of donor:acceptor. Due to segregated dopamine receptor subtype expression in MSNs [24], the directionality of cAMP response to dopamine efficiently identifies D_1_- and D_2_-MSNs [23]. Therefore, dopamine was first applied to neurons while cAMP was monitored in real-time, allowing for neuronal identification. After cAMP returned to the baseline, forskolin was then applied to measure the response. Absolute cAMP values were determined from the interpolation of a cAMP standard curve in permeabilized CAMPER neurons. Dopamine was added in phasic puffs, and forskolin was bath applied in the pH 7.2 recording buffer which consisted of: 1.3 mM CaCl_2_, 0.5 mM MgCl_2_, 0.4 mM MgSO_4_, 0.4 mM KH_2_PO_4_, 4.2 mM NaHCO_3_, 138 mM NaCl, 0.3 mM Na_2_HPO_4_, 5.6 mM D-Glucose, and 20 mM HEPES.

## 3. Results

### 3.1. Development and Validation of BiFC Screening Platform in a Neuronal Cell Model

**Figure 2:**
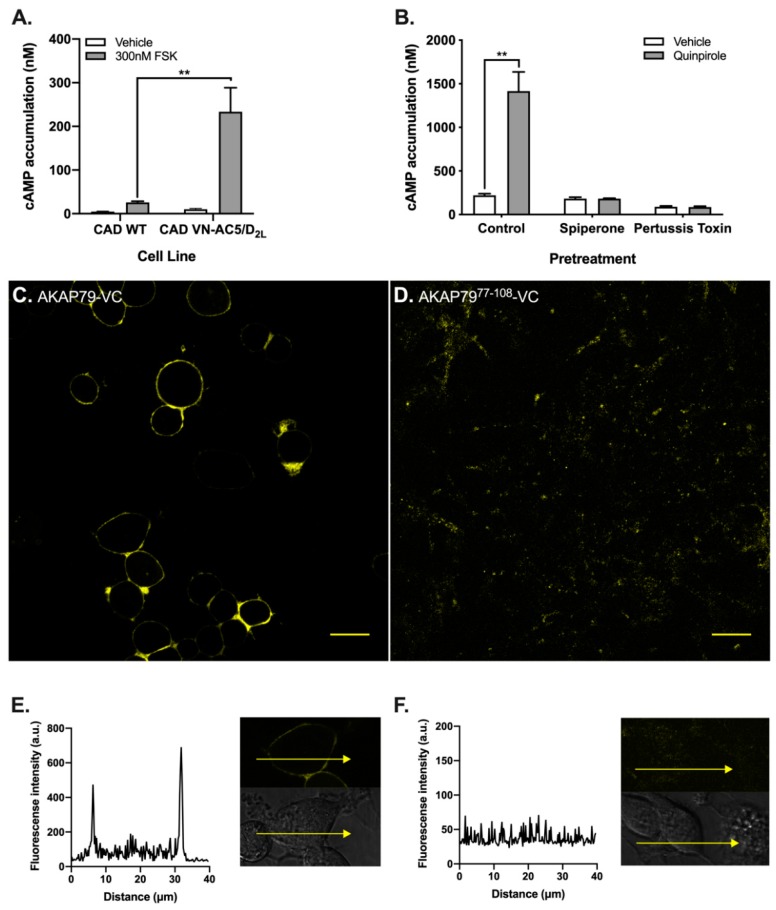
Functional activity profile of Cath a. differentiated (CAD) VN-AC5/D_2L_ cell line. Characterization of a stable cell neuronal model co-expressing the D_2L_ dopamine receptor as well as AC5 fused to the N-terminal BiFC fragment of Venus fluorescent protein (VN-AC5). The cAMP accumulation of the CAD VN-AC5/D_2L_ clonal cell line was significantly greater than the parental CAD WT cells following acute stimulation with 300 nM FSK (**A**). The CAD VN-AC5/D_2L_ cell line exhibited a 6.5-fold enhancement of cAMP accumulation upon forskolin (300 nM) stimulation following a 4-h treatment with quinpirole (3 μM) compared with vehicle treatment (**B**). Pretreatment with the D_2_ antagonist spiperone or pertussis toxin abolished the AC sensitization response. Statistical analysis (t-test) was performed, ** *p* < 0.01. Experiments (*n* = 3) were performed in duplicate with SEM shown. Positive and negative BiFC controls were used to validate the specificity and fluorescence intensity of the CAD VN-AC5/D_2L_ cell line. The 60x confocal images represented the CAD VN-AC5/D_2L_ transfected with 1 μg of (**C**) the positive control AKAP79-VC, where Venus fluorescence was clearly observed localized to the cell membrane or (**D**) the negative control AKAP79^77–108^-VC that exhibited no specific localization. Scale bars = 30 μm. Line scans of representative cells demonstrate an enhanced membrane-localized BiFC fluorescent signal for cells transfected with AKAP79-VC (**E**), compared with AKAP79^77–108^-VC (**F**).

We inserted the N-terminal Venus fluorescent protein BiFC fragment (VN155) as a fusion to the cytosolic N-terminal tail of human AC5 (i.e., VN-AC5). The VN-AC5 construct and the Gα_i/o_-coupled D_2_ dopamine receptor (D_2L_) were both stably expressed in Cath a. differentiated (CAD) cells. This CNS catecholaminergic cell line has been effectively used to visualize GPCR interactions in living cells and as a platform for multicolor BiFC analysis of receptor dimerization [25]. The selected CAD VN-AC5/D_2L_ clone was first evaluated for AC5 function by examining acute AC activity in response to 300 nM forskolin (FSK) (Figure 2A). The CAD VN-AC5/D_2L_ clone exhibited a significantly enhanced cAMP accumulation response over the CAD wildtype cells. Because the response of the CAD wildtype cells to FSK stimulation was 10% that of the CAD VN-AC5/D_2L_ clone, we could assume measured changes in cAMP accumulation reflect primarily AC5 activity.

The CAD VN-AC5/D2L clones were then evaluated for D2L function by examining D2 agonist-induced sensitization of AC activity. This paradoxical sensitized AC response occurs following persistent activation of most Gαi/o-linked receptors [7]. Examining cAMP accumulation in response to forskolin stimulation, following prolonged D2L agonism, allowed the assessment to D2-mediated heterologous sensitization of recombinant AC5. A four-hour pretreatment with the D2 agonist quinpirole to induce the sensitization response resulted in a robust increase to 1400 nM cAMP accumulation following stimulation with 300 nM forskolin, a 6.5-fold increase over the vehicle-treated cells (Figure 2B). Together, these results confirmed the presence of recombinant AC5 and D_2_-receptor-mediated heterologous sensitization. To further validate the requirement of the D_2_ receptor function, sensitization was measured in the presence of the D_2_ antagonist spiperone or following pertussis toxin treatment to inhibit Gα_i/o_ coupling. Indeed, the quinpirole-induced sensitization was blocked by pretreatment with the D_2_ antagonist spiperone or overnight pertussis toxin treatment, indicating that the sensitization was both receptor and Gα_i/o_-dependent, as previously described [26]. These results demonstrated that the N-terminal BiFC tag did not interfere with AC5 activity and the development of the AC sensitization response.

We next sought to assess the interaction specificity and the BiFC fluorescence intensity of the VN-AC5 construct. To accomplish this goal, we developed complementary BiFC constructs using A-kinase anchoring proteins (AKAPs), a class of known and previously characterized AC5 interacting partners. Specifically, AKAP79 has been shown to localize and directly interact with AC5 at the cell membrane. Mapping studies of the AC5-AKAP79 interaction interface have identified residues 77–108 of AKAP79 to be essential to their interaction [27]. Indeed, the deletion of this region (AKAP79^77–108^) has been shown to disrupt the AC5-AKAP79 interaction in living cell models [27].

To establish positive and negative AC5 interaction controls, we developed complementary BiFC constructs in which the complementary VC155 BiFC fragment, separated by a 15 amino acid flexible linker, was fused to the C-terminus of both AKAP79 and AKAP79^77–108^. Transfection of AKAP79-VC into the CAD VN-AC5/D_2L_ cell line produced a robust, membrane-localized BiFC fluorescence response (Figure 2C,E). As expected, the expression of AKAP79^77–108^-VC failed to produce a specific membrane-localized fluorescent signal, indicating a lack of interaction with VN-AC5 (Figure 2D,F). These results demonstrated that the VN-AC5 construct was expressed and could accurately detect and indicate BiFC complementation in the CAD neuronal model with AKAP79-VC, but not AKAP79^77–108^-VC, a construct missing regions critical for complex formation.

### 3.2. BiFC Screening of Human Brain cDNA Library Identified AC5 Interaction Networks Using Fluorescence-Activated Cell Sorting

To interrogate changes in the AC5 protein interaction network that occur in living cells under different conditions, we developed a high-throughput workflow to express a BiFC-tagged cDNA library, assess AC5 interactions by fluorescence, and then isolate these cells for gene identification using NGS (Figure 1). The foundation of this experimental workflow was in the development of a unique BiFC-tagged human brain cDNA library. By cloning the cDNA library into a custom BiFC vector, each complementary DNA sequence would be fused to the complementary VC155 BiFC fragment through a short flexible linker when translated to protein. For each screening trial, the library of approximately 1 million distinct cDNA-VC plasmids was transfected into the CAD VN-AC5/D_2L_ cell line followed by 12-h treatment with either vehicle or the D_2_ agonist quinpirole (3 μM) to induce D_2_-mediated heterologous sensitization. To identify and compare changes in the AC5 protein interaction network in the absence or presence of prolonged D_2_-receptor activation, the BiFC-positive cells from each condition were evaluated using fluorescence-activated cell sorting (FACS). The collected BiFC-positive cells were lysed, and their plasmid DNA was extracted. Sequential rounds of PCR were used to first amplify the cDNA insert of the BiFC plasmid, then DNA sequence barcodes were added, denoting the FACS run number and treatment condition. The indexed samples were pooled and sequenced using the Illumina MiSeq format. After trimming the primer and adapter sequences from the results, the resulting raw sequences were matched against a human reference genome.

From the three independent trials of cDNA library transfection and FACS, a list of genes from each treatment condition was compiled. We identified 1387 sequences from vehicle-treated cells that were matched to the human genome, and 1099 sequences from the quinpirole treatment group (Table S1). By narrowing our focus to protein-coding genes that reproduced by appearing in at least two of the three separate FACS trials, we identified 106 genes exclusive to the vehicle treatment, 48 genes were identified under the quinpirole treatment, and 59 genes showed overlap in both vehicle and quinpirole conditions (Table S2), for a total of 213 genes.

We found that this screening method, successfully, captured and identified multiple previously validated AC5 interacting partners. AKAP6, tetraspannin7, and PKCζ have all been previously characterized as AC5 interacting partners, capable of modulating signaling activity [14,28,29,30]. These results further verify that AC5 protein interaction networks are indeed captured by the present strategy. Importantly, they also suggest that the newly identified interactions represent biologically relevant protein networks.

### 3.3. Assessment of Condition-Dependent AC5 Interaction Networks

In an effort to narrow the focus of the 213 genes identified as putative hits from the AC5 activity screen, functional clustering and pathway analysis were used to select 20 genes unique to either the vehicle or quinpirole treatment groups for further evaluation based on their role in known signaling pathways. Due to the diverse nature of the selected genes, assessing their functional roles through traditional methods, such as dominant-negative or small molecule inhibition, would be an impossible task. Rather, we chose to efficiently explore the potential functional consequences of these target genes on AC5 signaling using siRNA knockdown in HEK-AC5/D_2L_ cells.

Transfection of pooled siRNA to knock down the expression of targeted genes were followed by functional assays to assess the effect of each gene on both acute and sensitization signaling of AC5 (Table S3). Most of the genes had a greater inhibitory effect on sensitized AC5 activity when compared to acute AC5 activity (Table S3). Five genes reduced both AC5 acute stimulation and sensitization responses by greater than 40% following siRNA knockdown (Table S3). Additionally, we found three genes that upon knockdown potentiated acute AC5 activity. We further validated twelve of those targets by examining the effect of the individual siRNA that made up the pooled sample used for the initial assessment (Table S4). Requiring at least two of the four individual siRNA to reproduce the functional effects of the pooled sample, we identified two genes of particular interest. The protein phosphatase 2A (PP2A) catalytic subunit beta (PPP2CB) was identified from the vehicle treatment group as a modest modulator of both acute and sensitized AC activity and NSF sensitive attachment protein alpha (NAPA) from the quinpirole treatment group as an apparent selective modulator of sensitized AC activity (Table S4).

### 3.4. Functional Characterization of AC5 Associated Proteins

The phosphorylation of AC5 has been shown to directly regulate its activity in a site-specific manner [31,32]. Therefore, the identification of a novel phosphatase interacting partner under vehicle treatment conditions could further reveal the mechanism by which AC5 maintains a consistent basal level of activity, as well as the dynamic regulation of activity in response to post-translational modifications [17,32,33]. Additionally, NAPA is known to play an important role in intracellular membrane trafficking and membrane fusion [34]. Furthermore, the activity of NAPA has been shown to be regulated by G-protein signaling events [35]. However, it remains unknown if prolonged Gα_i/o_-mediated signaling promotes the association of NAPA with AC complexes. Importantly, neither protein is currently recognized to associate with or modulate AC5 activity.

**Figure 3:**
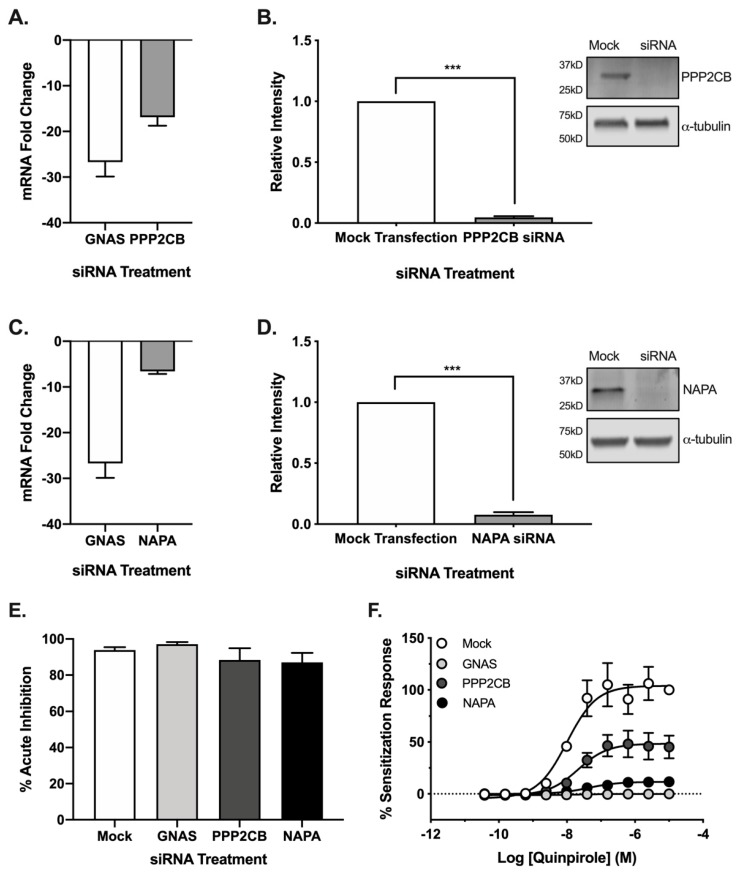
Gene knockdown does not alter acute D_2_ signaling but decreases the maximum efficacy of AC sensitization response in HEK-AC5/D_2L_ cells. Biochemical validation of siRNA knockdown was carried out by qPCR (**A**,**C**) and western blotting (**B**,**D**). Cell fractions from mock or siRNA-treated cells were subjected to western blot analysis using target antibodies for (**B**) PPP2CB and (**D**) NAPA, as well as listed loading controls. Data were standardized to mock transfection (left) and representative immunoblots (right). Statistical analysis was performed (t-test), *** *p* < 0.001. The ability of the Gα_i/o_-coupled D_2_ receptor to inhibit AC remained intact following gene knockdown (**E**). Quinpirole (3 μM) maximally inhibited the AC response to acute stimulation with 3 μM forskolin under all siRNA conditions. siRNA knockdown of target genes reduced the maximal efficacy of the AC sensitization response without affecting the potency of quinpirole (**F**). Experiments (*n* = 3) were performed in duplicate with SEM shown.

Having validated the functional effects of PPP2CB and NAPA siRNA knockdown, we next examined the role of these proteins on AC5 function following acute and chronic D_2_ receptor activation. Initial experiments confirmed gene knockdown following siRNA treatment by qPCR. siRNA knockdown of PPP2CB resulted in a 16-fold change in mRNA, while NAPA knockdown resulted in a 6-fold change (Figure 3A,C). Because changes in mRNA are not equal with regard to translation into proteins, analysis by western blot confirmed that siRNA knockdown significantly reduced (*p* ≤ 0.001) the protein expression for the genes PPP2CB and NAPA (Figure 3B,D) [36].

Although siRNA knockdown attenuated forskolin-stimulated AC5 activity (Table S3), neither PPP2CB nor NAPA knockdown altered the maximal efficacy of Gα_i/o_ modulation of AC5 activity (Figure 3E). We then tested the effect of knockdown on heterologous sensitization of AC5 following pretreatment with increasing concentrations of the D_2_ receptor agonist quinpirole. Gα_s_ (GNAS) siRNA was used as a positive control due to its known role in the development of sensitization [37]. These results revealed a marked reduction in maximal AC5 response, with no change in the potency of quinpirole (Figure 3F). The siRNA knockdown of NAPA expression resulted in a nearly 90% reduction of the sensitization response, while PPP2CB knockdown reduced the sensitization response by approximately 50%. Together, these results suggested that gene knockdown did not disrupt the potency or maximal efficacy of receptor-mediated Gα_i/o_ signaling, and the reduced acute and sensitization responses reflected changes in AC5 activity.

**Figure 4:**
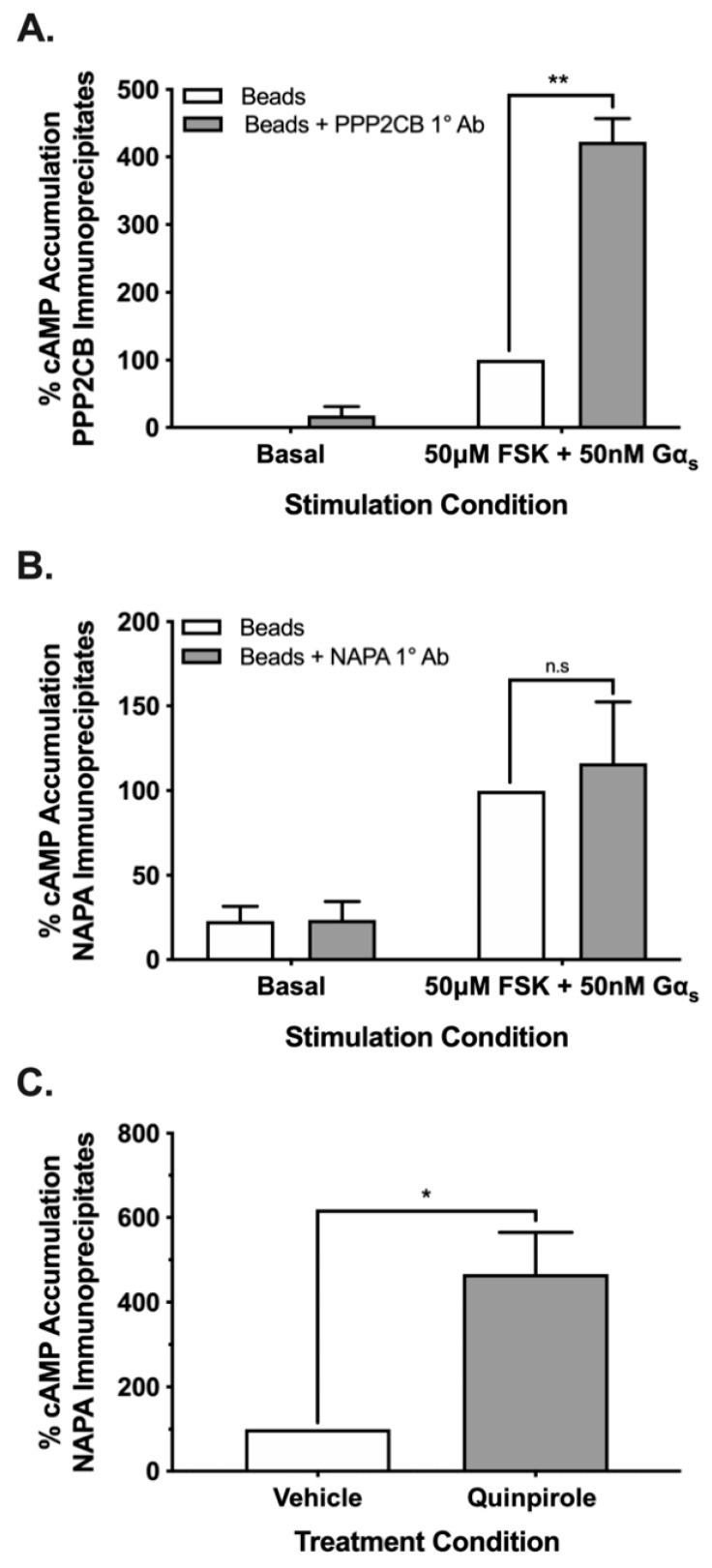
Adenylyl cyclase activity is elevated in immunoprecipitates of interacting partners. Immunoprecipitation of PPP2CB and NAPA resulted in enhanced AC activity in the pulldown product in a treatment-dependent manner. The PPP2CB immunoprecipitate exhibited 4-fold enhancement of AC activity under vehicle treatment conditions (**A**). The NAPA immunoprecipitate exhibited no significant difference in AC activity under vehicle treatment conditions (**B**). Quinpirole pretreatment resulted in a significant 4.5-fold enhancement of AC activity from NAPA immunoprecipitates compared to the vehicle-treated group (**C**). Statistical analysis was performed (t-test), * *p* < 0.05, ** *p* < 0.01. Experiments (*n* = 3) were performed in duplicate with SEM shown.

In an effort to further validate the association of PPP2CB and NAPA with AC5 complexes, we employed an orthogonal method using co-immunoprecipitation AC activity assays [27]. Endogenous PPP2CB or NAPA was immunoprecipitated from HEK AC5/D_2L_ cell lysates using their respective antibody, and AC activity was measured in the pulldown product. The PPP2CB immunoprecipitates were incubated with activated Gα_s_ and FSK, revealing significantly increased AC activity versus the control (Figure 4A). Because NAPA was observed in the AC5 BiFC screen exclusively following chronic quinpirole treatment, we preincubated the cells with a vehicle or quinpirole overnight prior to the immunoprecipitation assay. Stimulation of endogenous NAPA immunoprecipitates following vehicle treatment showed no significant increase in AC activity compared to the control (Figure 4B). In contrast, overnight treatment of cells with quinpirole resulted in a significant increase in the AC activity observed in endogenous NAPA immunoprecipitates compared with the vehicle-treated group (Figure 4C).

**Figure 5:**
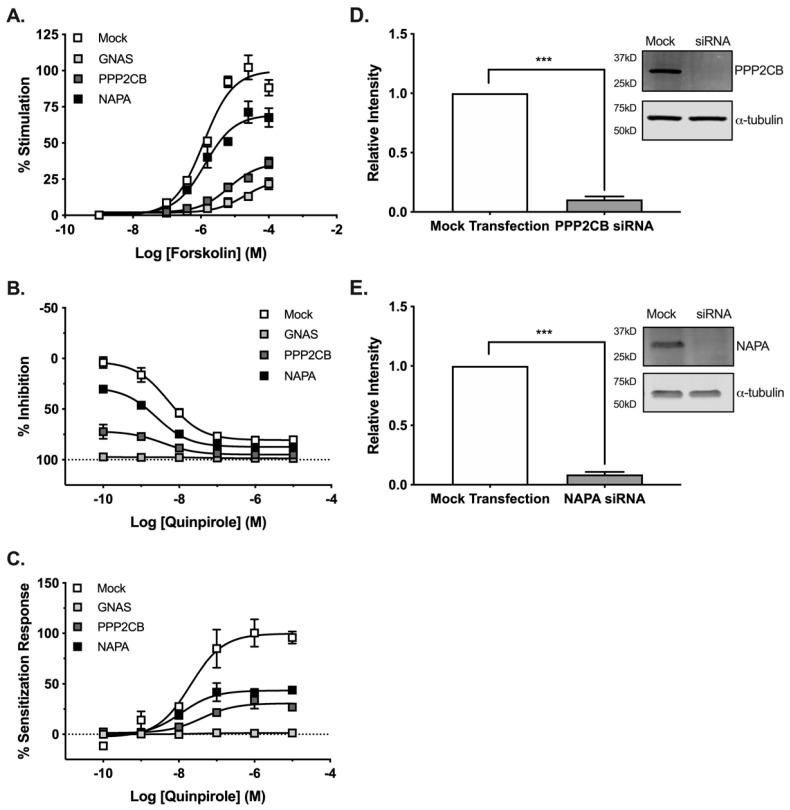
Target gene knockdown reduces maximal adenylyl cyclase activity in CAD VN-AC5/D_2L_ cells. siRNA knockdown of the target genes reduced the maximal forskolin-stimulated AC activity in CAD VN-AC5/D_2L_ cells (**A**). The ability of the Gα_i/o_-coupled D_2_ receptor to acutely inhibit AC activity remained intact following siRNA knockdown (**B**). siRNA knockdown of the target genes resulted in a reduction of the maximal sensitization response, with no difference in the potency of quinpirole to promote the development of sensitization (**C**). Experiments (*n* = 3) were performed in duplicate with SEM shown. Biochemical validation of siRNA knockdown was carried out by western blotting (**D**,**E**). Cell fractions from mock or siRNA-treated cells were subjected to western blot analysis using target antibodies for (**D**) PPP2CB and (**E**) NAPA, as well as listed loading controls. Data were standardized to mock transfection (left) and representative immunoblots (right). Statistical analysis was performed (t-test), *** *p* < 0.001.

We next sought to explore the potential for PPP2CB- or NAPA-AC5 complexes in neuronal models of AC5 signaling. Initially, we examined the effects of gene knockdown in the CAD-D_2L_/AC5 model used for the BiFC screening. siRNA knockdown of PPP2CB and NAPA reduced forskolin-stimulated AC activity by 75% and 25%, respectively (Figure 5A). In contrast, there was no effect of gene knockdown on the potency or maximal inhibitory activity of quinpirole (Figure 5B). Consistent with the HEK cell data, the siRNA knockdown of NAPA and PPP2CB expression both resulted in a significant reduction of the maximal sensitization response (*p* ≤ 0.05) (Figure 5C). Analysis by western blot confirmed that siRNA knockdown in CAD-D_2L_/AC5 cells significantly reduced (*p* ≤ 0.001) the protein expression of PPP2CB and NAPA (Figure 5D,E). Despite the effects on acute and sensitized adenylyl cyclase responses, knockdown of expression had no significant effect on the potency or maximal efficacy of D_2_ receptor-mediated Gα_i/o_ signaling.

**Figure 6:**
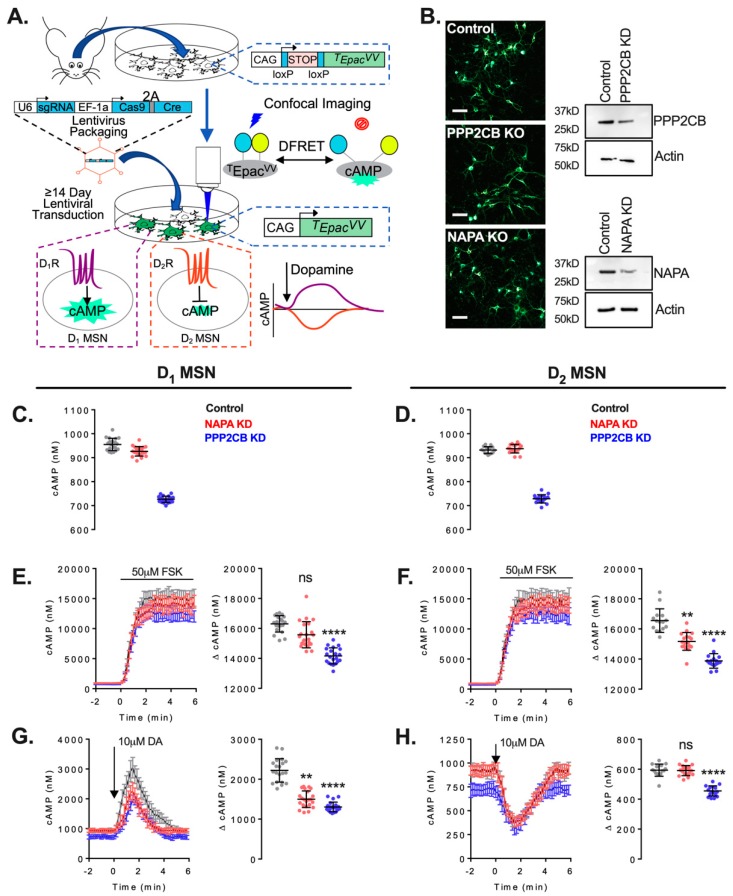
CRISPR/Cas9 deletion of NSF (N-ethylmaleimide-sensitive factor) attachment protein alpha (NAPA) and protein phosphatase 2A catalytic subunit (PPP2CB) reduces cAMP signaling in primary striatal neurons. (**A**) schematic of the experimental design. (**B**) (**left**) representative confocal images of CAMPER biosensor expressing neurons in DIV14 primary striatal neurons. Images are a merged stack of mTurquoise and Venus fluorescence. Scale bar equals 50 μm. (**B**) (**right**) western blot validation of CRISPR/Cas9-mediated knockdown of NAPA and PPP2CB. Baseline cAMP levels were significantly decreased in PPP2CB KO compared with control in both D_1_-MSN (**C**) and D_2_-MSN (**D**). D_1_-MSN cAMP dynamics following bath application of 50 μM forskolin revealed significantly reduced maximal response amplitude in PPP2CB KO compared with control (**E**). D_2_-MSN cAMP dynamics following bath application of 50 μM forskolin revealed significantly reduced maximal response amplitude in both NAPA and PPP2CB KO compared with control (**F**). D_1_-MSN cAMP dynamics following the phasic application of 10 μM dopamine revealed significantly reduced maximal response amplitude in NAPA and PPP2CB KO compared with control (**G**). D_2_-MSN cAMP dynamics following the phasic application of 10 μM dopamine revealed significantly reduced maximal response amplitude in PPP2CB KO compared with control. All statistics were nonparametric Kruskal–Wallis test, Dunn’s post-test, ** *p* < 0.01, **** *p* < 0.0001 in (**C**–**E**,**G**), and (**H**) or *p* = 0.0044 in (**F**).

We next sought to explore the potential role of PPP2CB and NAPA in cAMP signaling in native striatal neurons where endogenous AC5 is abundantly expressed [38]. These studies utilized a CRISPR/Cas9 gene-editing strategy in combination with real-time imaging of cAMP dynamics in CAMPER mice to examine dopamine and forskolin regulation of cAMP in the D_1_ receptor containing direct MSNs (dMSNs) and D_2_ receptor-expressing indirect MSNs (iMSNs) (Figure 6A) [23]. CRISPR/Cas9 reduction of PPP2CB and NAPA showed a decrease in protein expression in both neuron types (Figure 6B). The knockdown of PPP2CB was accompanied by a significant reduction in basal cAMP accumulation in both dMSNs and iMSNs, whereas NAPA reduction was without effect on basal cAMP signaling (Figure 6C,D). A reduction in forskolin-stimulated cAMP accumulation following PPP2CB knockdown was observed in both neuron types (Figure 6E,F). PPP2CB knockdown also reduced D_1_ receptor-stimulated cAMP accumulation in dMSNS (Figure 6G). The effects of NAPA reduction on the cAMP accumulation in response to forskolin or D_1_ receptor activation were generally more modest than that of PPP2CB reduction. Consistent with our HEK and CAD cellular models, the genetic knockdown of PPP2CB or NAPA did not alter the maximal level of D_2_ receptor-mediated inhibition of cAMP accumulation (Figure 6H).

## 4. Discussion

AC5 is the principal AC isoform expressed in striatal medium spiny neurons and controls neuronal activity through the dynamic integration of complex dopaminergic signals [2,39]. Acute and chronic dopamine signals can elicit differential modes of AC regulation, requiring unique protein network elements to regulate the spatial and temporal control of cAMP signaling within a cellular compartment and maintain homeostasis [12,13,14]. Furthermore, the duration of receptor activation influences the composition and localization of these dynamic regulatory complexes [15,16,17].

In an effort to understand how AC protein networks adapt to the complex integration of acute versus chronic dopamine receptor signaling, we presented the development and validation of a novel screening strategy to interrogate AC5-protein interaction networks in a high-throughput manner. Through the development of a neuronal cell model expressing an AC5-VN BiFC construct, we were able to screen a unique human brain BiFC-tagged cDNA library under varying treatment conditions. By combining FACS in alignment with NGS, it allowed for the rapid sequestration of BiFC-positive cells and the identification of the potential interacting partners unique to each treatment condition. This screening methodology successfully identified multiple previously characterized AC5 interacting partners, including AKAP6, tetraspanin 7, and PKCζ [14,28,29,30]. These interacting partners have all been demonstrated to modulate AC5 signaling activity, and moreover, were all identified through different capture methods, including immunoprecipitation, yeast two-hybrid, and co-purification. These results further demonstrate that functional AC5 protein interaction networks can be captured by the present strategy.

We hypothesized that the AC5 protein interaction network would differ under normal (vehicle) and sensitizing (quinpirole) conditions. Although we found several shared genes under both conditions, we had a large number of genes that appeared under the only vehicle or quinpirole treatment. Two of the genes from the BiFC screen were functionally validated in three unique cellular models. The fidelity of the observed effects involving PPP2CB and NAPA was striking, considering the diversity of the assay readouts and cellular systems. For example, the results using siRNA in transfected HEK and CAD cells functionally validated the roles of PPP2CB and NAPA in the modulation of AC5. These effects appeared specific to AC5 signaling as Gαi/o signaling was unchanged in both models. Furthermore, we demonstrated that the effects of gene knockdown were generally recapitulated in the MSNs from CAMPER mice. The conditional interactions from the BiFC screen were also supported by co-immunoprecipitation activity assays, demonstrating that PPP2CB associated with AC5 under vehicle conditions and NAPA associated with AC5 following quinpirole pretreatment [40]. These unique AC5 networks may be regulated by one or more AKAPs, as shown for inflammatory pain [40]. Together, these results suggest that PPP2CB and presumably PP2A is constitutively associated with AC5, whereas the involvement of NAPA appears to involve activity-dependent protein complex interactions.

Protein phosphorylation is a significant regulator of nearly all cellular processes, including enzyme activity, protein interactions, and intracellular localization [41,42]. A role for phosphorylation in regulating AC activity, including the heterologous sensitization response, has long been supported by evidence demonstrating that the enzymatic activity of AC isoforms can be directly regulated by phosphorylation in a site-specific manner [17,33,37]. Moreover, AC5 shows complex regulation by phosphorylation, including both positive and negative regulation of activity [17,32]. While phosphatases are not typically considered to be drivers of the phosphorylation state, cAMP regulation of phosphatase activity can result in the dynamic regulation of both phosphatase activity as well as substrate specificity, ultimately regulating the phosphorylation state of a target [41]. The protein phosphatase PP2A and AC5 have both been shown to interact with the intracellular protein scaffold AKAP79 [17]. Previous data has shown that PP2A regulatory subunits can be targeted and phosphorylated by PKA in a cAMP-dependent manner, which can subsequently increase the phosphatase activity of PP2A, as well as alter its substrate specificity [43]. Indeed, AC type 8 (AC8) has been shown to directly interact with a regulatory subunit of the PP2A heterotrimer [42].

Presently, our results indicated that PPP2CB was associated with the AC5 interaction network under basal conditions. That siRNA knockdown of PPP2CB reduced acute and sensitized AC activity that might reflect constitutive inhibition by PKA, which is a known negative regulator of AC5 activity [32,41]. While additional studies are necessary to address the possibility that manipulating PPP2CB expression could indirectly influence AC activity, future research may show that the prolonged Gα_i/o_-coupled receptor activation required for the development of the sensitization response could also disrupt the dephosphorylation of AC5 through changes in activity or substrate specificity, thereby enhancing the stimulatory effects of phosphorylation [43,44,45].

GPCRs are well known for their ability to upregulate or downregulate their presence at the cell membrane in response to extracellular stimuli. Following periods of prolonged inhibition, additional receptors can be trafficked from intracellular stores to the cell membrane to help compensate for the reduction in receptor signaling. Numerous studies have shown that GPCR interaction with N-ethylmaleimide-sensitive factor (NSF) contributes to the determination of receptor trafficking [46,47,48]. Through its interaction with NAPA, NSF binds both vesicle and target membrane SNARE proteins to form a complex that is essential to membrane fusion during intracellular trafficking and exocytosis [46,47,48]. We observed that the intracellular trafficking-associated protein, NAPA, preferentially associated with the AC5 interaction network following prolonged Gα_i/o_-mediated stimulation. Adenylyl cyclases are members of dynamic complexes of interacting partners, which localize signaling machinery to distinct locations within the cell to accommodate receptor signaling [49,50]. The association of AC5 with NAPA may represent a compensatory mechanism similar to that observed with GPCR desensitization. Specifically, prolonged Gα_i/o_-coupled receptor activation results in the trafficking of additional AC enzymes to the cell membrane and dramatically enhances agonist-stimulated cAMP accumulation.

We described the successful development and execution of a novel BiFC-based screening strategy to capture and identify novel protein networks through the use of FACS and NGS. We used this screening approach to overcome a persistent obstacle to identifying the AC5 protein interaction network associated with basal and sensitized AC5 activity. The screening results were validated through a series of functional experiments in two recombinant model systems exogenously expressing AC5. The findings were subsequently translated in a set of experiments using D_1_ and D_2_ MSNs that endogenously express AC5. Through this unbiased strategy, we identified and validated novel AC5 regulators that enhance our understanding of the striatal AC signaling network. Continued research to understand the molecular mechanisms for assembling the interaction networks will further our scientific understanding of adaptive pathological changes associated with neurological disorders.

## Supplementary Materials

The supplementary materials are available at https://www.mdpi.com/2073-4409/8/11/1468/s1.
